# Vitamin K enhances the production of brain sulfatides during remyelination

**DOI:** 10.1371/journal.pone.0203057

**Published:** 2018-08-27

**Authors:** Daniela C. Popescu, He Huang, Naveen K. Singhal, Leah Shriver, Jennifer McDonough, Robert J. Clements, Ernest J. Freeman

**Affiliations:** 1 Department of Biological Sciences, Geauga campus, Kent State University, Burton, Ohio, United States of America; 2 Department of Chemistry and Biology, University of Akron, Akron, Ohio, United States of America; 3 Department of Biological Sciences, Kent campus, Kent State University, Kent, Ohio, United States of America; Instituto Cajal-CSIC, SPAIN

## Abstract

Multiple sclerosis (MS) is a devastating neurological disease, which is characterized by multifocal demyelinating lesions in the central nervous system. The most abundant myelin lipids are galactosylceramides and their sulfated form, sulfatides, which together account for about 27% of the total dry weight of myelin. In this study we investigated the role of vitamin K in remyelination, by using an animal model for MS, the cuprizone model. Demyelination was induced in C57Bl6/J mice, by feeding them a special diet containing 0.3% cuprizone (w/w) for 6 weeks. After 6 weeks, cuprizone was removed from the diet and mice were allowed to remyelinate for either 1 or 3 weeks, in the absence or presence of vitamin K (*i*.*p*. phylloquinone, 2mg, three times per week). Vitamin K enhanced the production of total brain sulfatides, after both 1 week and 3 weeks of remyelination (*n* = 5, *P*-values were <0.0001), when compared with the control group. To determine whether or not there is a synergistic effect between vitamins K and D for the production of brain sulfatides, we employed a similar experiment as above. Vitamin K also increased the production of individual brain sulfatides, including d18:1/18:0, d18:1/20:0, d18:1/24:0, and d18:1/24:1 after 3 weeks of remyelination, when compared to the control group. In addition, vitamin D enhanced the production of total brain sulfatides, as well as d18:1/18:0, d18:1/24:0, and d18:1/24:1 sulfatides after 3 weeks of remyelination, but no synergistic effect between vitamins K and D for the production of total brain sulfatides was observed.

## Introduction

MS usually starts as a relapsing-remitting disease, which is believed to be caused by ineffective remyelination that consequently leads to axonal damage [1–3]. It is thought that a relapse involves demyelination, whereas remittance is associated with myelin repair (“remyelination”). The relapsing remitting MS disease most commonly advances over time toward progressive MS disease, which is probably caused by ineffective remyelination that consequently leads to axonal damage. Remyelination is important for the restoration of the rapid axonal impulse transmision and neuroprotection [1–2].

There are several experimental animal models that allow studying the potential mechanism of remyelination. Among them, the cuprizone-induced demyelination model is frequently used [4–6]. Cuprizone (Oxalic acid bis-cyclohexylidenehydrazide) is a copper chelator, which, when ingested by mice, causes copper deficiency and oligodendrocytes degeneration [7]. The mechanism of cuprizone neurotoxicity is not well defined, though. Cuprizone’s mode of action seems not to be only based on its copper-chelating properties by impairing the function of an enzyme in the electron transport chain, cytochrome-C oxidase, which has copper as a cofactor [7], but may also have a more complex action by impacting other cellular processes [8–9]. Myelin consists of water (40%) and dry mass (60%), and the dry mass of myelin has a high lipid content (>70%). The most abundant myelin lipids are galactosylceramides and their sulfated form, sulfatides [10], which together account for about 27% of the total dry weight of myelin [11]. Decreases in myelin sulfatides content in the brain have been implicated as important factors in the disruption of myelin stability and function [11–12]. Interestingly, brain sulfatide metabolism has been shown to be regulated by vitamin K, a fat-soluble vitamin and a positive correlation between the dietary vitamin K and brain sulfatides has been demonstrated [13], however, no studies investigated whether or not vitamin K has an impact on the brain sulfatides levels during remyelination. It is also worth mentioning that there have been inconsistencies in the literature regarding the effect of vitamin K depletion or supplementation on sulfatide levels [14]: while Sundaram et al. have shown that vitamin K increased the levels of brain sulfatides, an earlier study has shown no change in the levels of brain sulfatides, suggesting that vitamin K may have a role in maintaining constant levels of brain sulfatides [14].

Another fat-soluble vitamin, vitamin D, has been shown recently to stimulate remyelination [15]. Notably, although vitamin K and vitamin D have been demonstrated to display synergistic effects and increase the bone density in humans [16], no synergistic effects of vitamin K and vitamin D on intestinal calcium absorption, renal calcium reabsorption, and cancellous and cortical bone mass have been shown in young rats with hypocalcemia [17].

The main goal of this study was to investigate whether or not vitamin K increases the production of sulfatides during remyelination. To examine the effects of vitamin K on remyelination, we used the cuprizone-induced-demyelination model [18]. More specifically, we evaluated the impact of vitamin K on the levels of brain sulfatides during remyelination by using mass spectrometry and assessed the effects of vitamin K on the extent of remyelination of the corpus callosum, by using Black-Gold^®^ staining. We also determined the effects of vitamin D on the sulfatide levels and whether or not there is a synergistic effect of vitamins K and D on the levels of brain sulfatides during remyelination.

Although evidence suggests that vitamins K modulates myelination [11, 15, 19, 20], to the best of our knowledge this is the first study that unveils the impact of vitamin K on the production of sulfatides during remyelination, and may have significant relevance in finding new therapies that promote remyelination.

## Materials and methods

### Materials

We used a standard rodent diet (TD. 00588, which is the Teklad Global 18% Protein Rodent Diet, known as 2018), a 0.3% cuprizone-containing diet (TD. 140805, which contains as a base diet TD. 00588), a vitamin K deficient diet (TD. 97053) [21], a diet deficient in vitamins K and D (TD. 150653), a purified rodent diet (TD. 150654), a 0.3% cuprizone-containing diet (TD. 150655), and a vitamins K and D deficient diet containing 0.3% cuprizone (TD. 150675, which contains as a base diet TD. 150653). All diets were in the form of pellets and obtained from Harlan Laboratories, Inc., Indianapolis, IN. Vitamin K1 (10mg/mL) was obtained from Hospira, Inc., Lake Forest, IL, and Butler Schein, Dublin, OH. Calcitriol (1 μg/mL) was obtained from McKesson (PSS World Medical, Great Lakes), Leetsdale, PA. Sulfatides and cerebrosides standards were purchased from Avanti Polar Lipids, Inc., Alabaster, AL; α-Galactosylceramide was purchased from BioVision, Inc., Milpitas, CA; 25-Hydroxyvitamin D3 and Vitamin K2 (Menaquinone-4, MK-4) solutions were obtained from Sigma Aldrich, Inc., Saint Louis, MO. Black-Gold^®^ II (AG105) was purchased from Millipore, Billerica, MA.

### Experimental design

All animal experiments were approved by the Institutional Animal Care and Use Committee of Kent State University (protocol # 390 DP 14–16) with all appropriate effort made to minimize animal suffering. C57Bl6/J male mice (7 weeks of age) were ordered from *The Jackson Laboratory*, housed in plastic cages (4 mice per cage), and provided with *ad libitum* water and regular diet for one week of the acclimatization period, followed by various experimental dietary interventions, as described below.

After the first week of acclimatization, cuprizone was used to induce demyelination, by feeding mice (n = 70) a diet containing 0.3% cuprizone for 6 weeks. The food was changed three times a week, and mice were weighed weekly. The negative control group for the first demyelination experiment consisted of mice (n = 10) that were fed a regular diet and sacrificed after 6 weeks. These control mice were sacrificed together with 10 mice that were fed the cuprizone-containing diet. The rest of the mice (n = 56) were switched to either a regular diet (n = 18) or a vitamin K-deficient diet containing no cuprizone (n = 38). The mice switched to either a regular diet or a diet deficient in vitamin K recovered for either 1 or 3 weeks. The mice on the vitamin K-deficient diet were divided into two groups: 1) vitamin-K deficient diet with intraperitoneal (*i*.*p*.) saline injection (n = 19), and 2) vitamin-K deficient diet and *i*.*p*. vitamin K1 injection (2 mg, three times per week, n = 19). These mice were housed in cages with mesh floors to avoid coprophagy. To buffer the mice from the mesh floors, cylindrical mouse tunnels were used, and nestlets to have them gnaw on and make nests. 1 or 3 weeks after cuprizone removal, mice switched to either a regular diet (n = 18) or a vitamin K-deficient diet containing no cuprizone (n = 38), were sacrificed. Specifically, 9 mice per each group were sacrificed 1 week after cuprizone, and another 9 or 10 mice per group were sacrificed 3 weeks after cuprizone. 5 mice per experimental condition were used for lipidomics analysis, and 4 or 5 mice per experimental group were used for the Black-Gold^®^ staining or immunohistochemistry. All mice were anesthetized with isoflurane, prior to performing the intracardiac perfusion with 1xPBS, followed by 4% paraformaldehyde. The second cuprizone-induced demyelination experiment was performed in the same manner, with minor modifications: after being fed the 0.3% cuprizone containing diet for 5 weeks, the mice were switched to a 0.3% cuprizone containing vitamins K and D- deficient diet for 1 week, and then, the remyelination phase was started by switching the mice to either a regular diet or a diet deficient in both vitamins K and D with *i*.*p*. injections of phylloquinone (2 mg, four times a week), calcitriol (0.2 μg, twice weekly), both phylloquinone (2 mg, four times weekly), and calcitriol (0.2 μg, twice weekly) or normal saline.

### Lipidomics analysis

#### Tissue homogenization

Mouse brain tissues were transferred to 1.5 mL microcentrifuge tube, followed by the addition of 100 μL methanol and 200 μL water. Brain tissues were homogenized the by repeating following steps: (1) freezing in liquid nitrogen; (2) thawing in room temperature; (3) sonication. The brain homogenates were subsequently split into two equal amounts and placed in two 1.5 mL microcentrifuge tubes.

#### Metabolite and lipid extraction from brain tissue

Half of the mouse brain was placed in 1.5 mL micro-centrifuge tubes and extracted by a modified Bligh and Dyer extraction method. Proteins were denatured by adding 0.1 mL cold methanol. The brain tissues were then processed by three cycles of freezing in liquid nitrogen, thawing and sonication. Following cell lysis, 750 μL 1:2 (v: v) chloroform: methanol were added to the sample lysates, followed by 125 μL chloroform. After vortexing, 250 μL of water was added and samples were incubated at -20°C for one hour. The samples were then centrifuged at 1000 × g for 15 minutes at 4°C to give a two-phases: an aqueous layer on top, organic layer below, with a protein disk interface. The organic phase containing lipids was collected into another 1.5 mL micro-centrifuge tube and dried in a CentriVap Concentrator (LABCONCO, Kansas, MO, USA). Samples were normalized by protein concentration quantified with a Bicinchoninc Acid (BCA) protein assay (G-Biosciences, St. Louis. MO, USA). Samples were resuspended in chloroform: methanol: water (2:1:0.1 (v:v:v)) in a volume of 200 μL.

#### Quantitation of sulfatides levels

Porcine brain sulfatides were used as calibration curve standards (Avanti, Alabaster. AL) and dissolved into a solution of chloroform: methanol: water (2:1:0.1 (v: v: v)). This was subsequently diluted to the following concentrations: 0.25, 0.50, 1.25, 2.50, 5.00, 12.5, 25.0, 50.0, 125, 250 μg/mL. 5 μL of standards and extractions were subsequently analyzed with LC-MS/MS. Reversed phase liquid chromatography was performed on the same instrument at a flow rate of 30 μL/min using a Kinetex C18 column. The mobile phase for isocratic elution consisted of water: acetonitrile 50%: 50% with 5 mM NH_4_OAc for 40 min. Sulfatide species (d18:1/24:1, d18:1/18:0, d18:1/24:0 and d18:1/20:0) were acquired in negative mode. Ionspray voltage was -4500V, declustering potential (DP) was -100V and collision energy was -80V. The ion source nebulizer gas (GS1), heater gas (GS2) and curtain gas (CUR) were 16, 15 and 25 psi, respectively. Sulfatide species were identified by *m/z* = 97.0 corresponding to the HOSO_3_^−^ ion. Calibration curves were constructed by linear regression analysis of integrated peak areas versus sulfatide concentration.

#### Quantitation of galactosylceramide (GalCer) levels

α-Galactosylceramide (BioVision, Milpitas. CA) was used as a standard quantification and dissolved into a solution of chloroform: methanol: water (2:1:0.1 (v: v: v)). This was subsequently diluted to the following concentrations: 0.001, 0.010, 0.100, 1.000, 10.00, 100 μg/mL. Lipid samples used for the determination of sulfatide levels were subsequently analyzed by LC-MS/MS. Reversed phase liquid chromatography was performed on an Eksigent MicroLC 200 at a flow rate of 40 μL/min using a Kinetex C18 column (EVO 5um, 2.1 mm x 15 cm). The mobile phase A consists of HPLC-grade water (5mM NH_4_OAc) and mobile phase B consists of HPLC-grade acetonitrile with 5mM NH_4_OAc. GalCer was eluted with the gradient: 0 min 70%B, 10 min 70%B, 13 min 90%B, 25 min 90%B, 30 min 70%B, 38 min 70%B. GalCer species (C18:0, C20:0, C24:0 and C24:1) were acquired and their transitions (*m/z* 728.60 → 184.07, *m/z* 756.64 → 184.07, *m/z* 810.68 → 184.07 and *m/z* 812.70 → 184.07) were selected for SRM. Ionspray voltage was 5000V, declustering potential was 100V and collision energy was 40V. The ion source nebulizer gas (GS1), heater gas (GS2) and curtain gas (CUR) were 16, 15 and 25 psi, respectively. Calibration curves were constructed by linear regression analysis of integrated peak areas versus GalCer concentration.

### Mass spectrometry analysis of menaquinone-4 (MK4) levels in the brain

MK4 (vitamin K2) is a light sensitive compound; therefore, all samples were shielded from direct light.

#### MK4 extraction

MK4 was extracted with a heptane extraction method based on previous studies [22, 23]. 200 μL isopropanol and 500 μL heptane were added to the tissue, followed by 30 seconds of vortexing. Samples were then subjected to a two rounds of freeze/thaw cycles and sonication. After a one-hour incubation in -20°C, the samples were centrifuged at 16,000 g for 30 min at 4°C. The upper phase (organic phase containing MK4) was collected and transferred to a new 1.5 mL microcentrifuge tube, and the middle phase (aqueous phase) was collected separately. The protein pellet was saved for normalization by BCA assay. Both liquid phases were dried in a CentriVap Concentrator (LABCONCO, Kansas, MO, USA) at room temperature.

#### Quantitation of MK4 levels

An MK4 standard (Cerilliant, Sigma-Aldrich, TX) was used as to create a calibration curve for quantification of MK4 levels in brain. Standards were dissolved into a solution of chloroform: methanol: water (2:1:0.1 (v: v: v)) and subsequently diluted to the following concentrations: 0.10, 0.50, 1.00, 5.00, 10.0, 50.0 μg/mL. Heptane extracted organic samples were re-suspended in approximately 200 μL of same solvent depending on protein levels determined by BCA. 5 μL of standards and extractions were subsequently analyzed with LC-MS/MS. Reversed phase liquid chromatography was performed on an Eksigent MicroLC 200 at a flow rate of 40 μL/min using a Kinetex C18 column (EVO 5μm, 2.1 cm x 15 cm). The mobile phase A was HPLC-grade water (5mM NH_4_OAc) and mobile phase B was HPLC-grade acetonitrile (5mM NH_4_OAc). MK4 was eluted with the following gradient: 0 min 70%B, 10 min 70%B, 13 min 90%B, 25 min 90%B, 30 min 70%B, 38 min 70%B. MK4 was acquired in positive mode on a TripleTOF 5600+ (SCIEX) mass spectrometer. A product ion scan using the transitions *m/z* = 445.32 → 427.31 and *m/z* = 445.32 → 363.24 to identify vitamin K. Ionspray voltage was 5000V, declustering potential (DP) was 80V and collision energy was 12V. The ion source nebulizer gas (GS1), heater gas (GS2) and curtain gas (CUR) were 16, 15 and 25 psi, respectively. Calibration curves were constructed by linear regression analysis of integrated peak areas versus MK4 concentration.

### Black-Gold^®^ staining

Free-floating brain sections of mice were washed with 0.9% NaCl, incubated for 12 minutes with pre-warmed 0.3% Black-Gold solution at 60°C, washed twice with saline, fixed for 3 minutes in 1% sodium thiosulfate, washed for three times with saline and mounted using glycerol.

### Immunohistochemistry for myelin oligodendrocyte glycoprotein (MOG)

Immunostaining for MOG was performed as previously described [24]. Briefly, free-floating coronal brain sections were washed three times with 1xPBS, incubated in 10% Triton X-100 with 1% H_2_O_2_ for 30 min, washed three times with 1xPBS, and blocked with 5% normal donkey serum (NDS) for 1 hour at room temperature. Myelin was detected by incubating sections overnight at 4°C with myelin oligodendrocyte glycoprotein (MOG) antibody (rabbit, 1:2,000; Abcam, Cambridge, MA) in 2% NDS. Sections were washed, incubated for 1 hr with a biotinylated donkey anti-rabbit secondary antibody (1:1,000; Jackson ImmunoResearch Laboratories, Inc., West Grove, PA) followed by incubation with an avidin-biotin complex (ABC reagent, 1:1,000; Vector Laboratories, Burlingame, CA). Sections were washed and incubated in diaminobenzidine (DAB kit, Vector Laboratories).

### Quantification of Black-Gold^®^-stained and MOG immunostained brain sections

Due to the variability of labeling that can occur with Black-Gold^**®**^ and DAB staining, all images used for quantification were compared with their respective control and the brain sections were labeled at the same time, and imaged under identical conditions. All images of the Black-Gold^**®**^-stained and MOG immunostained corpus callosum (from the midline to below the cingulum) were acquired using 10x objectives on a Leica microscope with an Optronics Magnafire^**®**^ digital camera. After we selected the area of interest, which included the corpus callosum between the midline and below the apex of the cingulum, we quantified the pixel intensities values by using NIH’s *ImageJ* software (http://rsb.info.nih.gov/ij/). We estimated the relative intensity of Black-Gold^**®**^ staining and MOG immunostaining in the corpus callosum as a percentage relative to the one of the control mice, which was given the value of 100.

### Statistical analyses

All statistical analyses were performed using the GraphPad Prism, *GraphPad 7*.*0 Software*, La Jolla, CA, either two-way ANOVA with Tukey’s multiple comparisons tests or unpaired t-tests. Data represent the means ± SEM. The correlation between MK4 and total sulfatides levels was computed by using GraphPad Prism.

## Results

### Feeding a cuprizone-containing diet induced weight loss during demyelination, and stopping the cuprizone diet resulted in weight gain during remyelination

**Fig 1:**
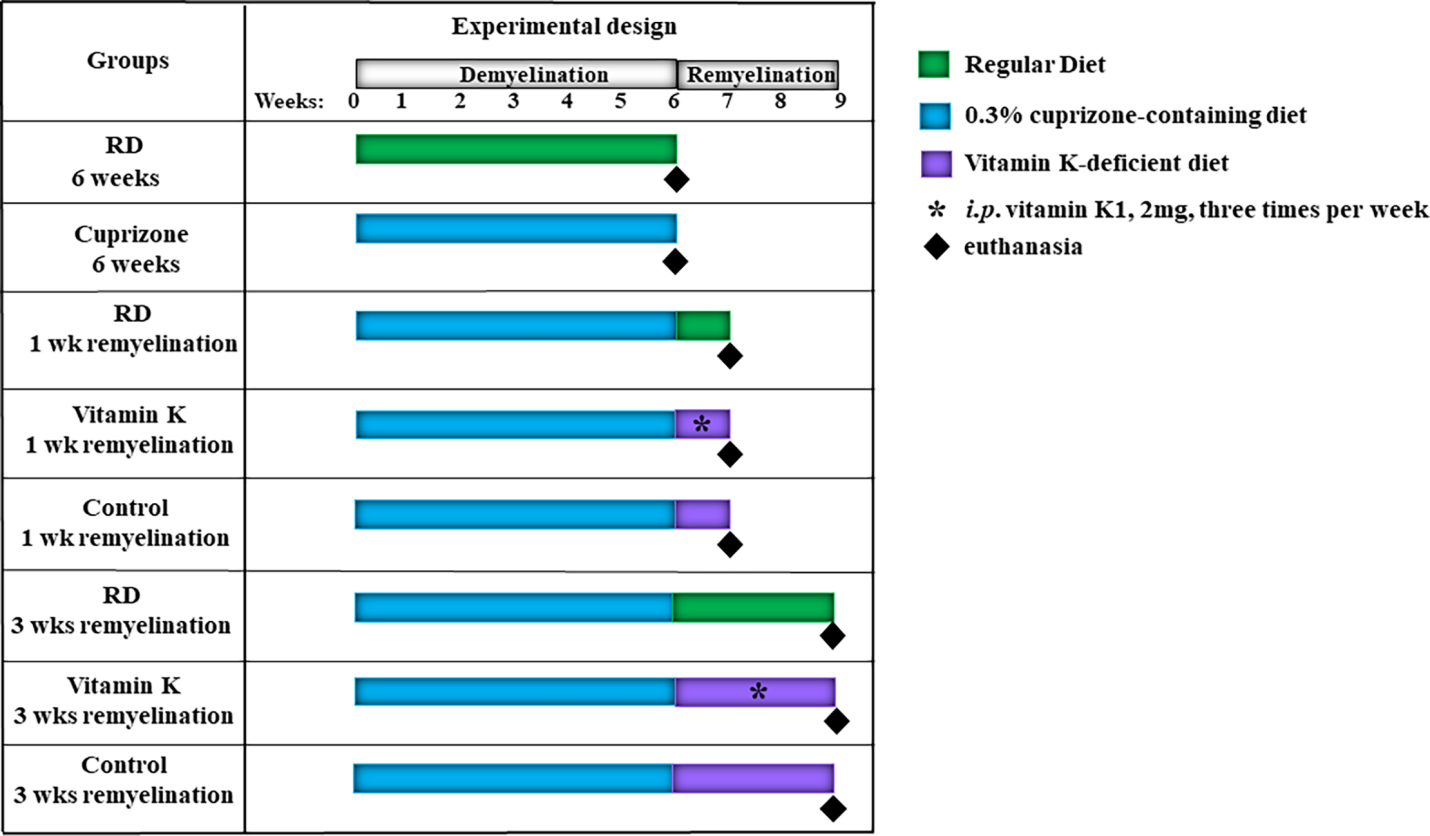
Diagram showing the experimental design described under the *Materials and Methods* section.

As previously shown, during the demyelination phase (**Fig 1**), the mice that were fed a 0.3% cuprizone-containing diet for 6 weeks lost weight when compared to the ones that were fed a regular diet (**Table 1**), and they started to gain weight during the remyelination phase, when the mice were switched to either a regular diet, or a diet deficient in vitamin K with either vitamin K injections or saline injections (**Table 2**).

**Table 1:** The mean body weight of mice during the demyelination phase. Mice were fed either a regular diet (Regular Diet, *n* = 10) or a diet containing 0.3% cuprizone (Cuprizone, *n* varied between *n* = 56 and *n* = 70) for 6 weeks. Shown values are mean ± SEM.

Week	Regular Diet	Cuprizone
**Week 0**	21.6 ± 0.33	21.71 ± 0.20
**Week 1**	22.5 ± 0.37	17.14 ± 0.16
**Week 2**	24.1 ± 0.23	16.19 ± 0.20
**Week 3**	25.3 ± 0.26	16.55 ± 0.16
**Week 4**	26 ± 0.42	17.14 ± 0.16
**Week 5**	26.85 ± 0.48	17.02 ± 0.17
**Week 6**	27.5 ± 0.42	19.6 ± 0.19

**Table 2:** The mean body weight of mice during the remyelination phase. The mice that were fed the 0.3% cuprizone-containing diet for 6 weeks, were switched to either a regular diet (Regular Diet, *n* = 18), a vitamin K-deficient diet with *i*.*p*. phylloquinone injections, 2 mg, three times a week (Vitamin K, *n* = 19), or a vitamin K-deficient diet with *i*.*p*. saline injections (200 μl, three times a week, Control, *n* = 19). Values are mean ± SEM.

Week	Regular Diet	Vitamin K	Control
**Week 0**	19.41 ± 0.42	19.63 ± 0.27	19.73 ± 0.31
**Week 1**	23.08 ± 0.29	23.36 ± 0.26	22.9 ± 0.32
**Week 2**	24.5 ± 0.23	26.5 ± 0.43	24 ± 0.39
**Week 3**	24.5 ± 0.17	27 ± 0.44	24 ± 0.5

### Vitamin K enhanced the production of brain sulfatides during the remyelination phase

**Fig 2:**
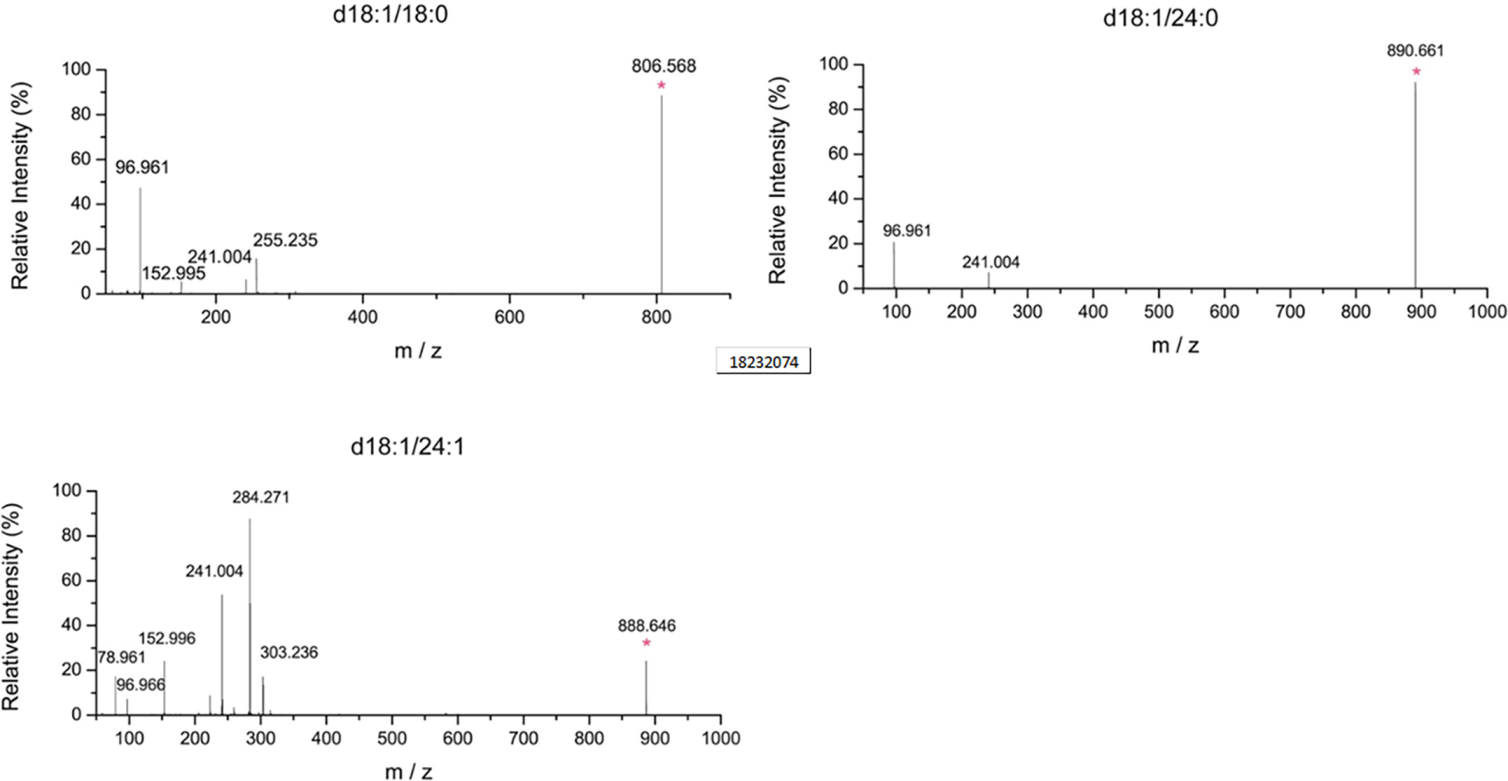
The transitions used by the mass spectrometry analysis for the determination of sulfatides levels. MS/MS data were collected by a product ion scan from the sulfatide standard and used to identify transitions for the determination of sulfatide levels in tissues. All spectra display a characteristic m/z = 97 which corresponds to the HOSO3^−^ ion.

The levels of various sulfatide isoforms, including d18:1/18:0, d18:1/24:0, and d18:1/24:1, as well as of the total levels of sulfatides in the brains isolated from mice that were fed the diets mentioned above, were determined by mass spectrometry. The MS/MS data that were used to determine the transitions for the determinations of sulfatides in the brains are shown in **Fig 2**.

**Fig 3:**
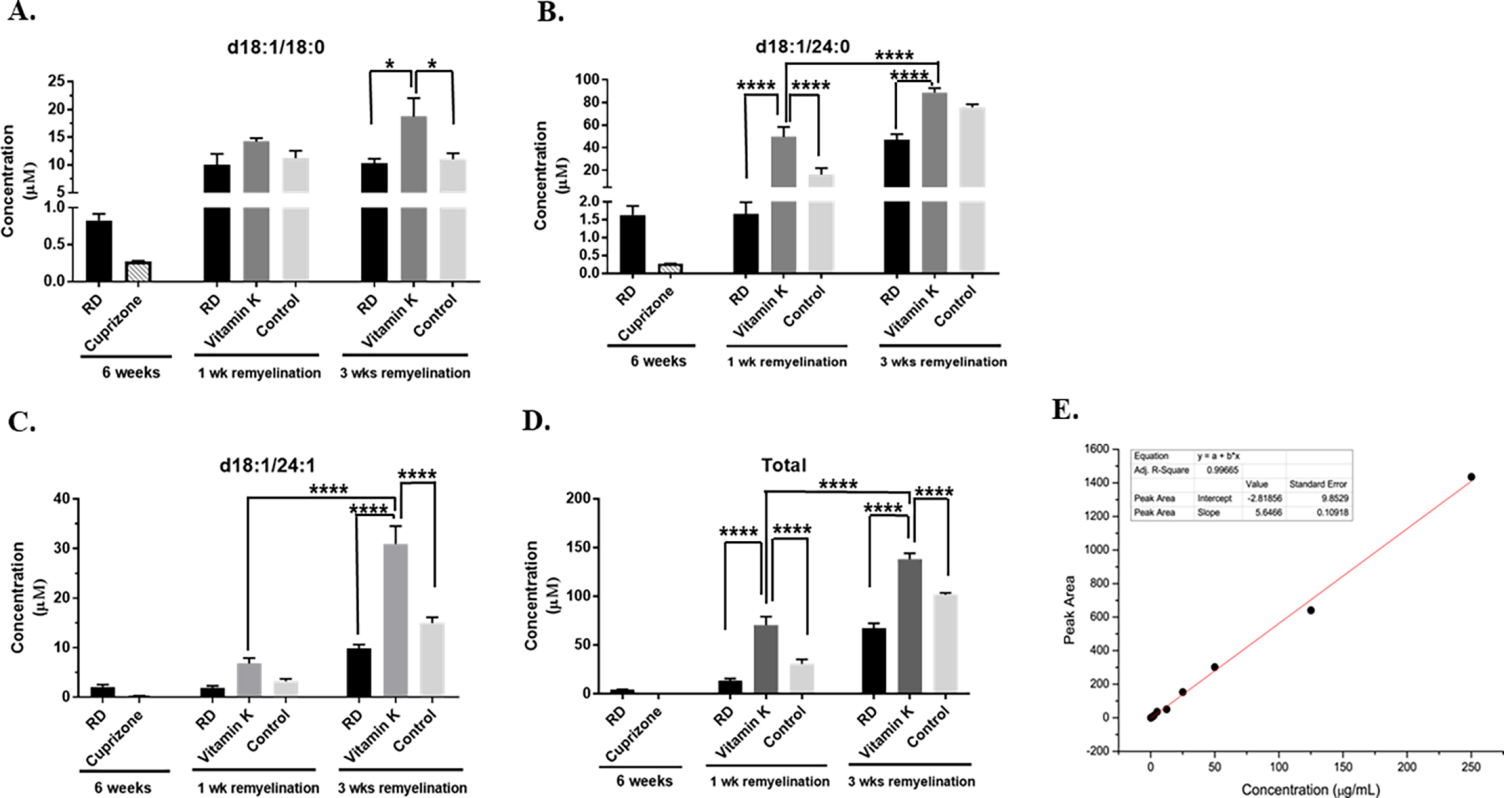
Vitamin K enhances the production of brain sulfatides. The levels of individual species of sulfatides, as well as the total sulfatides levels were determined by mass spectrometry analysis. Sulfatide levels were determined in brains isolated from mice that were fed the following diets: a 0.3% cuprizone- containing diet for 6 weeks (Cuprizone), regular diet for 6 weeks (RD), cuprizone for 6 weeks followed by 1 week remyelination on a regular diet (RD, 1 wk remyelination), cuprizone for 6 weeks followed by 1 week remyelination on a vitamin K-deficient diet with vitamin K injections (phylloquinone, 1mg/10g of body weight, three times a week, Vitamin K, 1wk remyelination), cuprizone for 6 weeks followed by 1 week remyelination on a vitamin K- deficient diet with saline injections (Control, 1wk remyelination), cuprizone for 6 weeks followed by a regular diet for 3 weeks (RD, 3 wk remyelination), cuprizone for 6 weeks followed by 3 weeks of remyelination on a vitamin K-deficient diet with vitamin K injections (1 mg/ 10 g body weight, Vitamin K, 3 wk remyelination), and cuprizone for 6 weeks followed by 3 weeks of remyelination on a vitamin K–deficient diet with saline injections (Control, 3 wk remyelination). Sulfatides were quantified with LC-MS/MS using linear regression analysis of integrated peak area versus the concentration of a sulfatide standard. Individual species of d18:1/18:0 (**A**.), d18:1/24:0 (**B**.), d18:1/24:1 (**C.**) sulfatides, and the total concentration of the sulfatide species (**D**.) are shown. n = 5 mice per group, and concentration (μM) is expressed as mean ± SEM. *P < 0.05, **P < 0.01, ***P < 0.001, ****P < 0.0001. Please see the text for the actual *P-values*. **E.** Calibration curve used for the determination of sulfatide concentration in mice brains. The calibration curve was constructed using standards diluted to the following concentrations: 0.25, 0.50, 1.25, 2.50, 5.00, 12.5, 25.0, 50.0, 125, 250 μg/mL.

The concentration of d18:1/18:0 sulfatide (**Fig 3A**) was increased after 3 weeks of remyelination in the brains of the mice that received vitamin K during remyelination, when compared to either the mice that remyelinated on a regular diet or to the control mice, which did not receive vitamin K (*n* = 5, *P*-values were 0.0141 and 0.0304, respectively). The concentration of d18:1/24:0 sulfatide (**Fig 3B**) was increased after 1 week of remyelination in the mice that received the vitamin K treatment when compared to either the mice that remyelinated on a regular diet or the control group (*n* = 5, *P*-values were <0.0001). Also, the levels of d18:1/24:1 sulfatide (**Fig 3C)** were increased in the brain samples of mice that received the vitamin K treatment and were allowed to remyelinate for 3 weeks, when compared to the ones that remyelinated on a regular diet or to the control mice (*n* = 5, *P*-values were <0.0001). Moreover, the total sulfatides levels in the brains were increased at both 1 week, as well as 3 weeks of remyelination in the mice receiving vitamin K (**Fig 3D**). They were significantly increased when compared to either the mice that remyelinated on a regular diet (*n* = 5, *P*-values were <0.0001) or to the control mice that remyelinated on a vitamin K-deficient diet (*n* = 5, *P*-values were <0.0001). Interestingly, vitamin K increased the levels of d18:1/24:0, d18:1/24:1, and total brain sulfatides after 3 weeks of remyelination, when compared to the ones of the mice that remyelinated for 1 week (*n* = 5, *P*-values were <0.0001, **Fig 3B**, **3C** and **3D**). The calibration curve that was used to determine the concentration of sulfatides in the brain samples is shown in **Fig 3E**.

#### Vitamin K treatment did not enhance the remyelination of the corpus callosum

**Fig 4:**
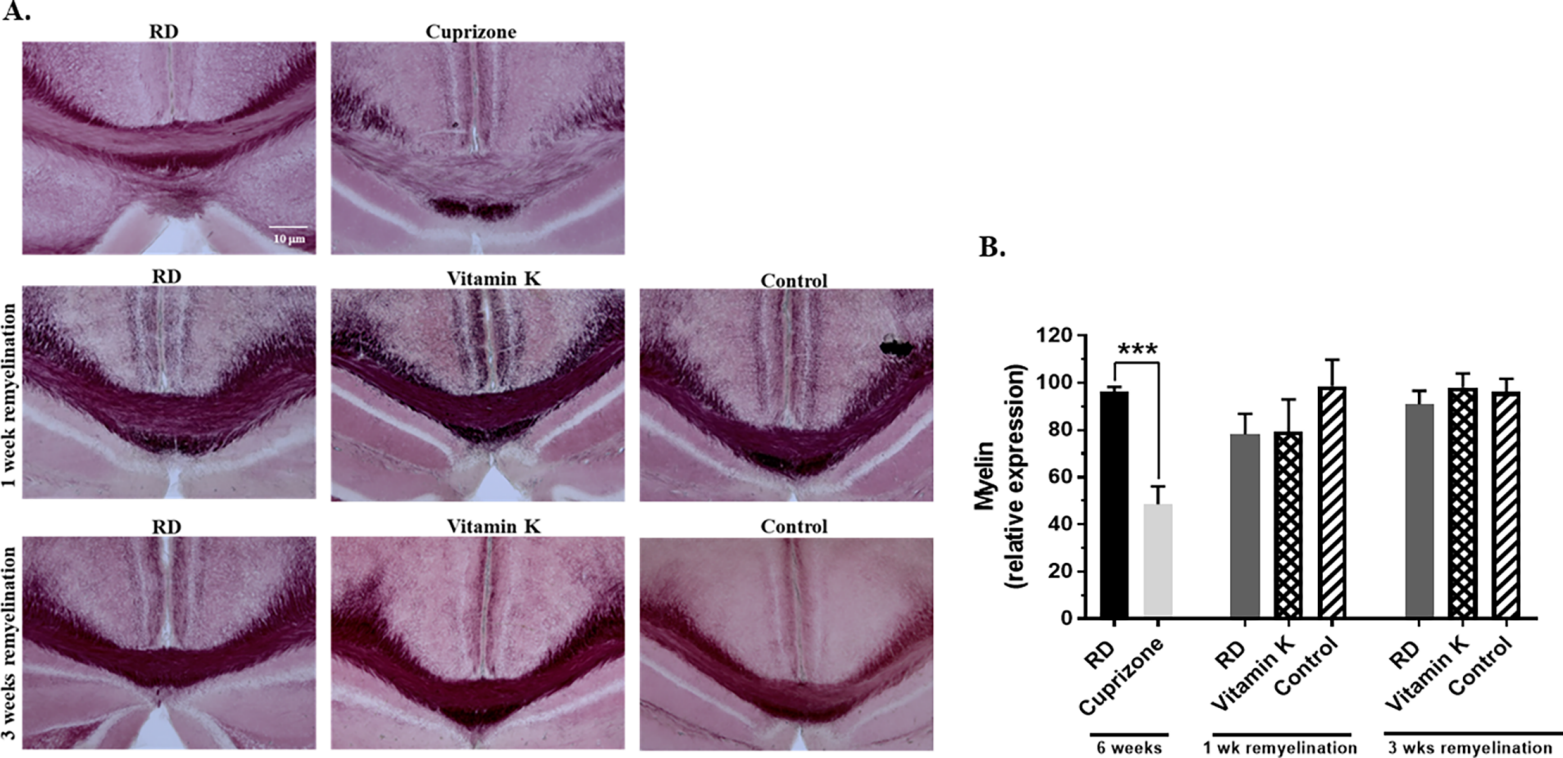
Vitamin K treatment does not enhance the remyelination of the corpus callosum. **A.** Representative brain coronal sections stained for myelin by using Black-Gold. **B.** The relative myelin expression of the corpus callosum was analyzed by quantifying the Black-Gold stained brain sections. As described in *Materials and Methods* section, all images used for quantification were compared with their respective control, and labeled at the same time and imaged under identical conditions. The area of the corpus the corpus callosum from the midline to below the cingulum was used to quantify the pixel intensities values using the *ImageJ* software (http://rsb.info.nih.gov/ij/). We estimated the relative intensity of Black-Gold staining in the corpus callosum as a percentage relative to the one of the control mice, which was given the arbitrary value of 100. The values are expressed as mean ± SEM. ***P < 0.001.

Because previous studies suggest that vitamin K modulates myelination [11, 20] we evaluated the impact of vitamin K on the remyelination of the corpus callosum, by using Black-Gold^**®**^ staining (**Fig 4**) and myelin oligodendrocyte glycoprotein (MOG) immunostaining (data not shown). Brain coronal sections were stained for myelin by using Black-Gold^**®**^ and the relative expression of myelin was quantified as described in the *Materials and Methods*, by using at least 2 sections per mouse and at least 3 mice per experimental group. While myelin relative expression was reduced by 49.5% (n = 4, *P-value* = 0.0004) in the corpus callosum of mice that were fed the 0.3% cuprizone-containing diet for 6 weeks when compared to the mice that were fed the regular diet for 6 weeks, there were no differences in the myelin relative expression during the remyelination phase in the mice that were switched to either a regular diet or a vitamin K-deficient diet, with or without vitamin K injections at neither 1 week nor 3 weeks of remyelination (**Fig 4B**). Similar results showing no change in remyelination in the vitamin K treated mice were obtained when the brain sections were MOG-immunostained and the MOG immunoreactivity was quantified (S1 Fig).

### Vitamin D enhanced the production of total brain sulfatides after 3 weeks of remyelination, but no synergistic effect between vitamins K and D for the production of total brain sulfatides was observed

**Fig 5:**
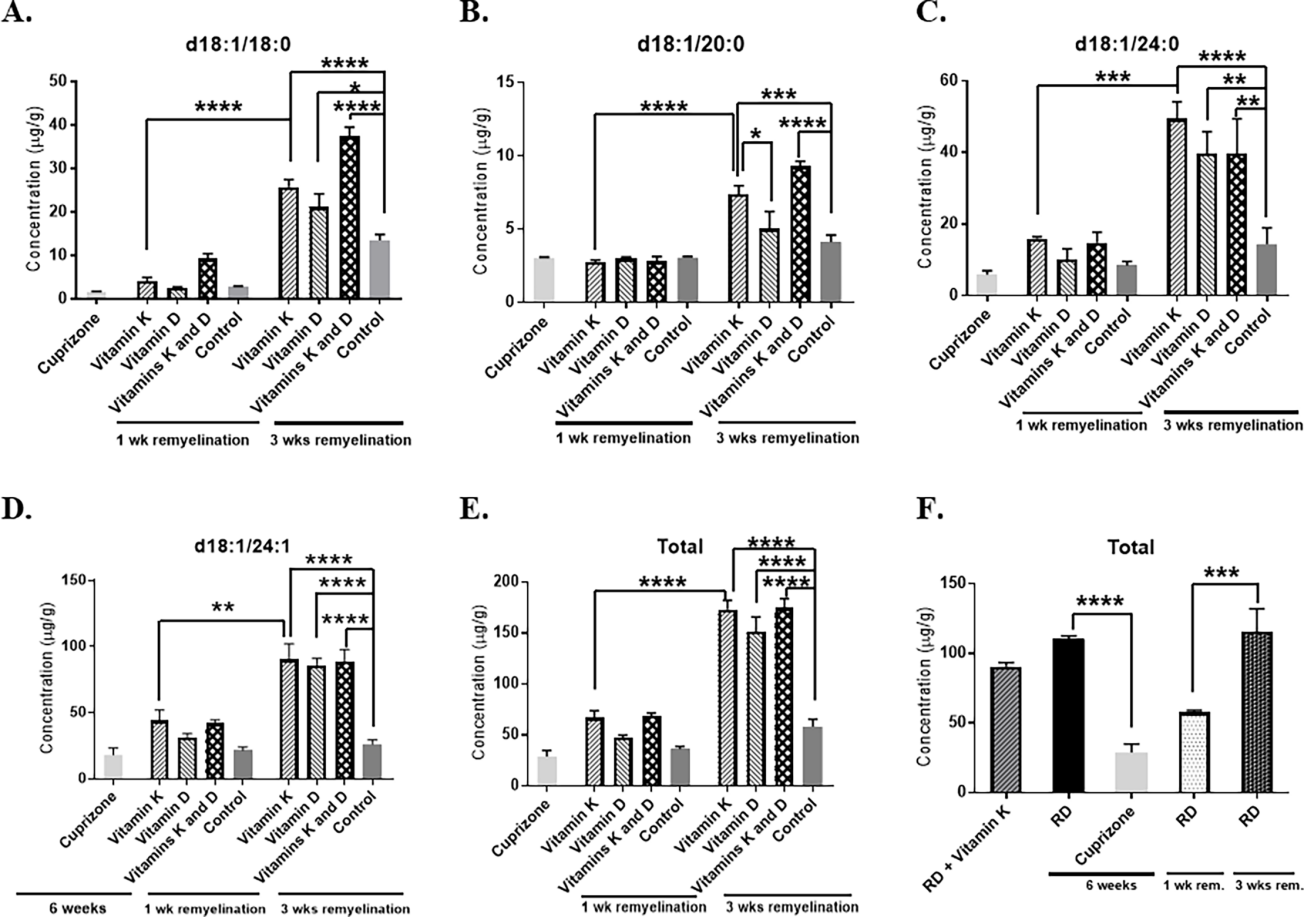
There is no synergistic effect between vitamin K and vitamin D on the synthesis of total brain sulfatides, but both vitamin D and vitamin K enhance the production of sulfatides during remyelination. The levels of individual species of sulfatides, as well as the total brain sulfatide levels were determined by mass spectrometry. Sulfatide levels were determined in brains isolated from mice that were fed various diets: a regular diet for 6 weeks (RD), a 0.3% cuprizone- containing diet for 6 weeks (Cuprizone), regular diet with vitamin K injections for 3 weeks (RD + Vitamin K), cuprizone for 6 weeks followed by 1 week remyelination on a regular diet (RD, 1 wk remyelination), cuprizone for 6 weeks followed by 1 week remyelination on a vitamins K and D- deficient diet with vitamin K injections (*i*.*p*. phylloquinone, 2mg, four times a week, Vitamin K, 1 wk remyelination), cuprizone for 6 weeks followed by 1 week remyelination on a vitamins K and D- deficient diet with vitamin D injections (*i*.*p*. calcitriol, 0.2 μg, twice weekly, Vitamin D, 1 wk remyelination), cuprizone for 6 weeks and 1 week remyelination on a vitamins K and D- deficient diet with vitamin K and vitamin D injections (Vitamins K and D, 1 wk remyelination), cuprizone for 6 weeks and 1 week remyelination on a vitamins K and D- deficient diet with saline injections (Control, 1 wk remyelination), cuprizone for 6 weeks and regular diet for 3 weeks (RD, 3 weeks remyelination), cuprizone for 6 weeks and 3 weeks of remyelination on a vitamins K and D deficient diet with vitamin K injections *(i*.*p*. phylloquinone, 2mg, four times a week, Vitamin K, 3 wks remyelination), cuprizone for 6 weeks followed by 3 weeks remyelination on a vitamins K and D- deficient diet with vitamin D injections (calcitriol, 0.2 μg, twice weekly, Vitamin D, 3 wks remyelination), cuprizone for 6 weeks + 3 week remyelination on a vitamins K and D- deficient diet with vitamin K and vitamin D injections (Vitamins K and D, 3 wks remyelination), and cuprizone for 6 weeks + 3 weeks remyelination on a vitamins K and D–deficient diet with saline injections (Control, 3 wks remyelination). Sulfatides were quantified with LC-MS/MS using linear regression analysis of integrated peak area versus the concentration of a sulfatide standard. Individual species d18:1/18:0 (**A**), d18:1/20:0 (**B**), d18:1/24:0 (**C**), d18:1/24:1 (**D**), and total concentration of the sulfatide species (**E** and **F**) are shown. *n* = 4 mice per group, and concentration (μg/g of brain tissue) is expressed as mean ± SEM. *P < 0.05, **P < 0.01, ***P < 0.001, ****P < 0.0001. Please see the text for the actual *P-values*.

Next, we investigated the impact of vitamin D on the production of brain sulfatides, and whether or not there is a synergistic effect between vitamin K and vitamin D on the production of brain sulfatides. Vitamin D (1, 25-dihydroxyvitamin D_3_ or calcitriol) has been shown previously to enhance remyelination [15], but to the best of our knowledge we are the first to evaluate the impact of vitamin D on the production of brain sulfatides and to test if there is a combined effect between vitamin K and vitamin D in terms of the production of brain sulfatides during remyelination. A similar experimental paradigm, as described previously, was used. Briefly, acute demyelination was induced by feeding mice a diet containing 0.3% cuprizone for 5 weeks. To ensure that the mice will become vitamin K and vitamin D deficient, we started the mice on a deficient diet earlier, during the last week of cuprizone-induced demyelination, when the mice were switched to a diet deficient in both vitamins K and D and containing 0.3% cuprizone. Next, the mice were switched to either a regular diet or a diet deficient in both vitamins K and D. The mice that were fed the vitamins K and D-deficient diet received either *i*.*p*. saline, vitamin K, vitamin D, or vitamins K and D injections and the levels of brain sulfatides **(Fig 5)** were analyzed by mass spectrometry at either 1 week or 3 weeks of remyelination.

Interestingly, all individual sulfatide species that were tested, including d18:1/18:0 (*n* = 4, *P*-value <0.0001, **Fig 5A**), d18:1/20:0 (*n* = 4, *P*-value = 0.0006, **Fig 5B**), d18:1/24:0 (*n* = 4, *P*-value <0.0001, **Fig 5C**), and d18:1/24:1 (*n* = 4, *P*-value <0.0001, **Fig 5D**) were significantly increased after 3 weeks of remyelination, in the brain samples of mice that received vitamin K (Vitamin K- 3 wks remyelination), when compared to the brain samples of mice that remyelinated for 3 weeks on a vitamins K and D- deficient diet (Control- 3 wks remyelination, **Fig 5A**–**5D**). Moreover, the total sulfatides levels in the brains of the animals that received either vitamin K (*n* = 4, *P*-value <0.0001) or vitamin D (*n* = 4, *P*-value <0.0001), as well as the ones that received both vitamins K and D injections (*n* = 4, *P*-value <0.0001) were greatly increased after 3 weeks of remyelination (**Fig 5E)** and significantly different when compared to the mice that remyelinated on a vitamins K and D deficient diet (Control) for 3 weeks. In addition, the levels of d18:1/18:0 (**Fig 5A**), d18:1/24:0 (**Fig 5C**), and d18:1/24:1 (**Fig 5D**) were significantly increased after 3 weeks of remyelination (*n* = 4, *P*-values were 0.0118, 0.0060, and <0.0001, respectively) in the brain samples of mice that remyelinated for 3 weeks and received vitamin D (Vitamin D- 3 wks remyelination), when compared to the brain samples of mice that remyelinated for 3 weeks on a vitamins K and D deficient diet (Control- 3 wks remyelination). Similarly, the levels of d18:1/18:0 (*n* = 4, *P*-value <0.0001, **Fig 5A**), d18:1/20:0 (*n* = 4, *P*-value <0.0001, **Fig 5B**), d18:1/24:0 (*n* = 4, *P*-value = 0.0061, **Fig 5C**), and d18:1/24:1 (*n* = 4, *P*-value <0.0001, **Fig 5D**) were significantly increased after 3 weeks of remyelination in the brain samples of mice that received both vitamin K and vitamin D (Vitamins K and D- 3 wks remyelination), when compared to the control mice.

As previously reported in the literature [13], vitamin K injections in healthy adult mice that were kept on a regular diet (RD + Vitamin K, **Fig 5F**) did not exhibit increased sulfatide levels in the brain, when compared to the ones of the healthy adult mice that were kept on a regular diet and did not receive any other treatments (RD-6 weeks). Although the levels of total brain sulfatides were increased at 3 weeks of remyelination when both vitamins K and D were used, and each of these vitamins: vitamin K, as well as vitamin D increased the levels of total brain sulfatides after 3 weeks of remyelination, no synergistic effect between vitamin K and vitamin D was noted (**Fig 5E**). Similarly, when both vitamins K and D have been used, no significant synergistic effects for individual sulfatides including d18:1/20:0, d18:1/24:0 and d18:1/24:1 were observed (**Fig 5B, 5C and 5D**). A synergistic effect was observed after 3 weeks of remyelination for the d18:1/18:0 sulfatide (**Fig 5A**).

### Determining the vitamin K levels in the brain

**Fig 6:**
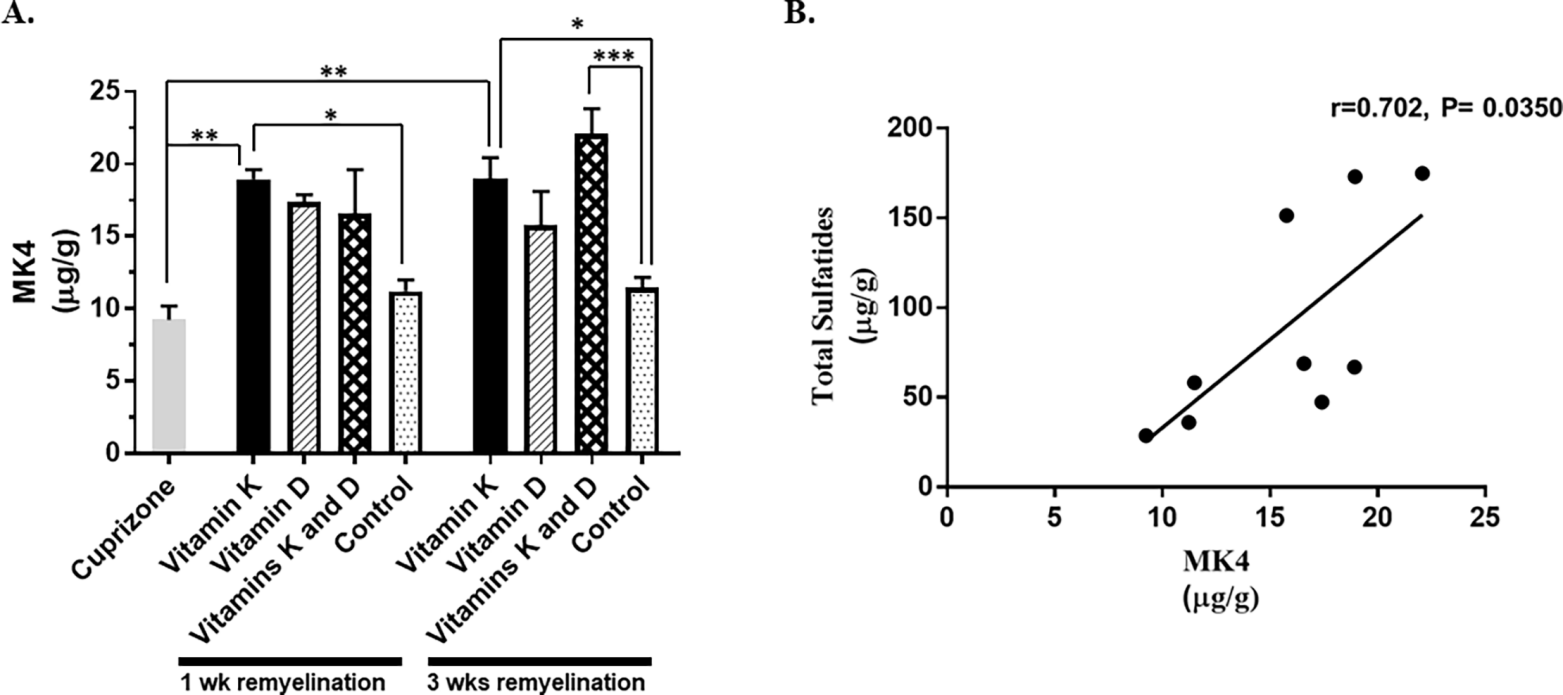
The vitamin K (MK-4) levels in the brain. **A.** The levels of MK-4 in the brain samples were determined by mass spectrometry *(n* = 4 brains per experimental condition), as described in the *Materials and Methods* section. Calibration curves were constructed by linear regression analysis of integrated peak areas versus MK4 concentration. Concentration (μg/g of brain tissue) is expressed as mean ± SEM.*P < 0.05, **P < 0.01, ***P < 0.001. Please see the text for the actual *P-values*. **B**. There was a significant positive correlation between the levels of MK-4 in the brain and the concentration of the total brain sulfatides (r = 0.67, *P-value* = 0.017). Each data point is expressed as mean (n = 4 per each experimental group).

Since vitamin K enhanced the production of sulfatides in the brain, next, we employed mass spectrometry to detect the levels of vitamin K (menaquinone-4, MK-4) in the brain. MK-4 (vitamin K2) is a metabolite of phylloquinone (vitamin K1), which was shown previously to accumulate in the brain [18–19]. We detected increased levels of MK-4 in the brain (**Fig 6A**). The levels of MK-4 in the brain samples of the mice that received *i*.*p*. phylloquinone injections as described under the *Materials and Methods* section, were statistically significant increased after both 1 week or 3 weeks of remyelination when compared to the control group that remyelinated on a vitamins K and D-deficient diet (*n* = 4, *P*-values were 0.0169 and 0.0221, respectively, **Fig 6A**). We found a positive correlation (r = 0.702, *P-value* = 0.035) between the levels of brain MK-4 and the total brain sulfatides content (**Fig 6B**).

Interestingly, the levels of MK-4 in the brain of the mice that were kept on the cuprizone diet for 6 weeks were decreased. This was probably due to a decreased volume of distribution for MK-4, which is a lipid-soluble vitamin. The volume of distribution decreased because cuprizone demyelinated the brains, decreasing their lipid content.

We have also measured the levels of vitamin D in the plasma by using mass spectrometry (data not shown), and we obtained similar results to the ones previously reported by Nystad *et al* [15].

### Vitamin K decreases the levels of brain galactosylceramides after 3 weeks of remyelination

**Fig 7:**
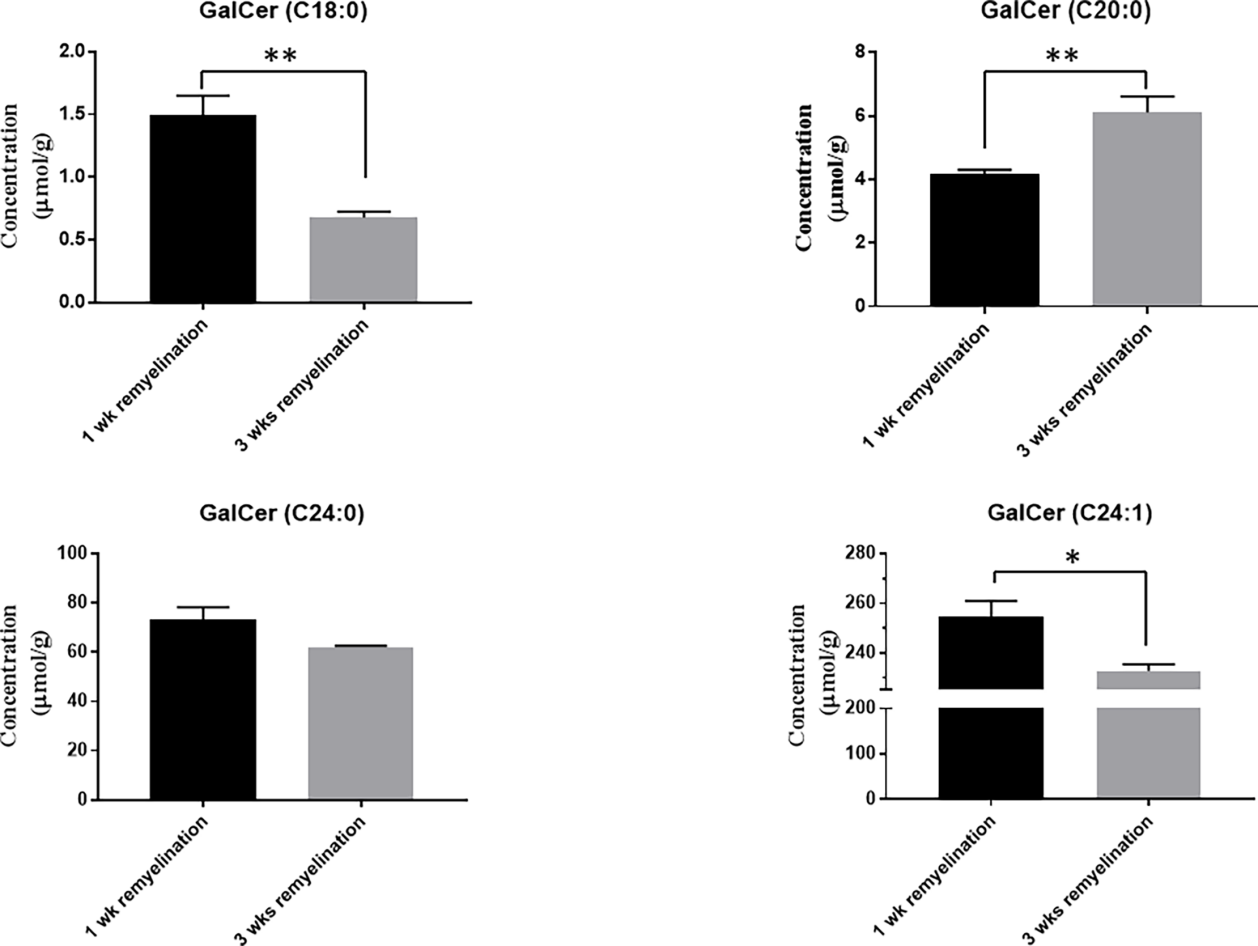
The levels of individual species of galactosylceramides, GalCer, during remyelination in the presence of vitamin K, were determined by mass spectrometry. The remyelination took place for either 1 week or 3 weeks in the presence of vitamin K. The levels of brain GalCer (C18:0) (**A**), GalCer (C20:0) (**B**), GalCer (C24:0) (**C**), and GalCer (C24:1) (**D**) were measured in the brain samples of mice that remyelinated for either 1 week or 3 weeks while receiving vitamin K injections as described under the *Materials and Methods* section. *n* = 4 mice per group, and concentrations are expressed as mean ± SEM. *P < 0.05, **P < 0.01. Please see the text for the actual *P-values*.

Since we determined that vitamin K enhances the production of brain sulfatides after 1 week and 3 weeks of remyelination and it is known that brain sulfatides are generated from galactosylceramides (GalCer or cerebrosides), next we measured the levels of individual species of brain galactosylceramides in the mice that remyelinated for either 1 week or 3 weeks in the presence of vitamin K (*(i*.*p*. phylloquinone, 2mg, four times a week, as described previously in *Materials and Methods*, **Fig 7**). Interestingly, the levels of GalCer (C18:0) and GalCer (C24:1) were decreased by vitamin K after 3 weeks of remyelination (*n* = 4, *P*-values were 0.0027 and 0.0185, respectively, **Fig 7A and 7D**), when compared with the ones of the mice that remyelinated for 1 week in the presence of vitamin K. There was also a tendency of GalCer (C24:0) levels to decrease after 3 weeks of remyelination in the presence of vitamin K, when compared with the brain samples of the mice that remyelinated for 1 week (**Fig 7C).** These data suggest that when vitamin K is present, as the remyelination progresses and the production of d18:1/18:0, d18:1/24:0, and d18:1/24:1 sulfatides increases, the levels of GalCer (C18:0), GalCer (C24:0), and GalCer (C24:1) decrease because the galactosylceramides are used to produce sulfatides.

The brain levels of GalCer (C20:0) in mice that remyelinated for 3 weeks *versus* those that remyelinated for 1 week while receiving vitamin K injections were increased (*n* = 4, *P*-value = 0.0091, **Fig 7B**).

## Discussion

In this study, we used cuprizone to induce a reversible acute demyelination of the brain, and demonstrated that vitamin K treatment increases the levels of brain sulfatides during remyelination, when there is a period of active myelination.

**Fig 8:**
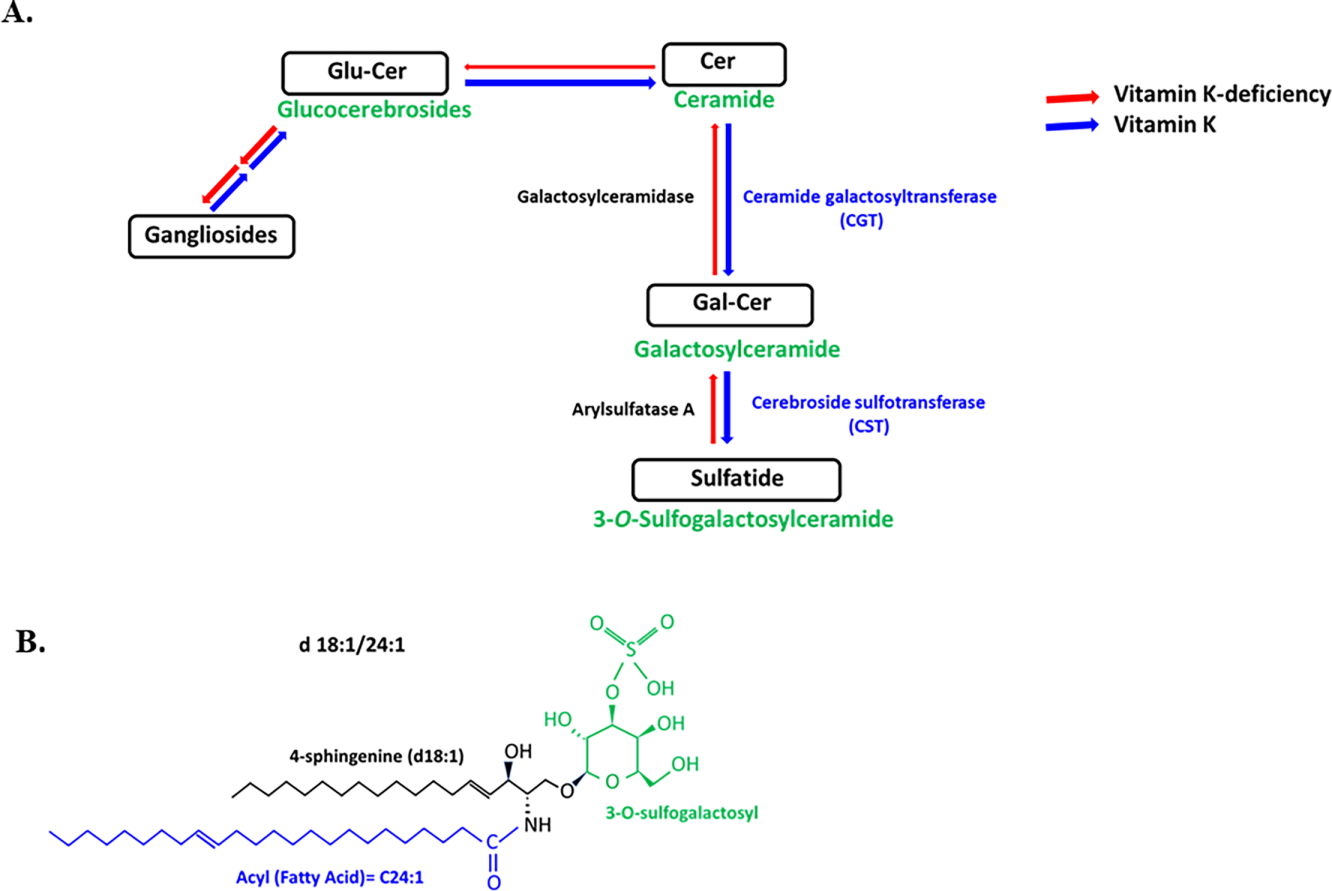
**A. A schematic diagram showing the synthesis of sulfatides from ceramide, and how the production of sulfatides during remyelination may be affected by a vitamin K-deficiency. B. The structure of d18:1/C24:1 sulfatide, which is a major myelin sulfatide.** Vitamin K increases the synthesis of this sulfatide, among other sulfatides, after 3 weeks of remyelination. This sulfatide is composed of a ceramide moiety containing 4-sphingenine (d18:1) with a C24:1 fatty acid.

Cerebroside sulfotransferase (CST) is the enzyme responsible for the production of sulfatides in the brain by transferring a sulfate group to Galactosylceramide (GalCer, **Fig 8A**). Our data indicate that vitamin K has an impact on the activity of CST during the active myelination process that occurs during the remyelination phase in adult mice, however, it does not affect CST activity in healthy 14 weeks-old adult mice (**Fig 5F**), in which the myelination process has already been completed. Previous studies showed that the activity of CST and production of brain sulfatides increased when young mice (16 day old) were exposed to vitamin K, whereas the administration of vitamin K to 1 month old mice did not significantly enhance the activity of CST [13]. Interestingly, CST has been previously shown to be a developmentally controlled enzyme that has a maximum activity in 18- to 22- days old mice, when the maximum myelination occurs [13]. To the best of our knowledge, our study is the first to unveil the impact of vitamin K on sulfatide production in adult mice, mice that completed developmental myelination, but underwent an acute demyelination, followed by remyelination.

Sulfatides display various structures, depending on the different lengths of the fatty acid chain (**Fig 8B)** and ceramide moiety [25]. Some major sulfatides are composed of ceramides containing 4-sphingenine (d18:1) with C24 fatty acids including C24:0 and C24:1 (d18:1/24:0 and d18:1/24:1), whereas some minor sulfatides including d18:1/18:0 and d18:1/20:0, are composed of ceramides containing d18:1 with C18 and C20 fatty acids [25]. Our data indicate that vitamin K enhances the production of both major and minor sulfatides during remyelination. When the remyelination progressed from 1 week to 3 weeks in the presence of vitamin K, the production of d18:1/24:1 increased (*n* = 5, *P*-value <0.0001, **Fig 3C**, and *n =* 4, *P*-value = 0.0011, **Fig 5D**) and the levels of GalCer (C24:1) decreased (*n =* 4, *P*-value = 0.0185, **Fig 7D)**, suggesting that the GalCer (C24:1) pool diminishes in the brain, since GalCer (24:1) is the precursor of d18:1/24:1 (**Fig 8**). Similarly, when the remyelination progressed from 1 week to 3 weeks in the presence of vitamin K, the production of d18:1/24:0 increased (*n* = 5, *P*-value <0.0001, **Fig 3B**, and *n =* 4, *P*-value = 0.0001, **Fig 5C**), and there was also a tendency of GalCer (C24:0) levels to decrease after 3 weeks of remyelination on vitamin K (**Fig 7C)**. Vitamin K also enhanced the production of other sulfatides, including d18:1/18:0 (n = 4, *P-value* <0.0001, **Fig 5A**) and d18:1/20:0 (n = 4, *P-value* <0.0001, **Fig 5B**). Interestingly, the levels of GalCer (C18:0) decreased, as expected (*n =* 4, *P*-value = 0.0027, **Fig 7A**), however, the concentration of GalCer (C20:0) increased (n = 4, *P-value* = 0.0091, **Fig 7B**).

Together, these data suggest a prioritized CST action on specific galactosylceramides, including the ones with 24:1 and 24:0 fatty acids, to increase the production of sulfatides with long chain fatty acid moieties, since these sulfatides are major components of myelin that may play important roles in myelin stability and maintenance [11]. Vitamin K also decreased the concentration of GalCer (C18:0) and consequently increased the concentration of brain d18:1/18:0, which is a sulfatide known to be present in neurons in small amounts and whose function is not known [25, 26].

Because vitamins K and D have been shown to display synergistic effects in some tissues [16], we tested if there are any synergistic effects between the effects of vitamins K and D on the production of sulfatides. We used the same regimen of calcitriol (*i*.*p*. 0.2 μg injections, twice weekly), that was previously used by Nystad et al. [15]. In our study, we showed that vitamin D also increased the production of two individual sulfatide species, which are typically associated with myelin, including d18:1/24:0 (*n* = 4, *P*-value = 0.0060, **Fig 5C**), and d18:1/24:1 (*n* = 4, *P*-value< 0.0001, **Fig 5D)**, after 3 weeks of remyelination. Vitamin D also enhanced the production of d18:1/18:0 sulfatide, after 3 weeks of remyelination (*n* = 4, *P*-value = 0.0118, **Fig 5A).** We were not surprised to learn that vitamin K and vitamin D did not display a synergistic effect on enhancing the brain sulfatide levels, since vitamin K and vitamin D play numerous physiological roles through a variety of organ specific mechanisms. Interestingly, although there are studies showing that vitamin K plus vitamin D increase the bone density in humans, other studies have shown no synergistic effects of vitamin K and vitamin D on intestinal calcium absorption, renal calcium reabsorption, and cancellous and cortical bone mass in young rats with hypocalcemia [17]. We also need to emphasize that the effects of vitamin K on bone physiology is mediated through osteocalcin, which is a protein that becomes γ-carboxylated with the help of vitamin K as a cofactor, whereas in the brain, the role of vitamin K in the sphingolipid metabolism is independent of its classic role as a cofactor for the γ-carboxylase enzyme. To the best of our knowledge this is the first study to show that vitamin K and vitamin D enhance the production of sulfatides during remyelination, with no synergistic effects.

Vitamin K1 (phylloquinone) is the dietary form of vitamin K, which is converted in the tissues to a form of vitamin K2 called menaquinone-4 (MK-4). Although vitamin K is present in the majority of the extrahepatic tissues as phylloquinone and MK-4, vitamin K in the brain occurs predominantly (>98%) as MK-4 [13, 19]. In our study we used vitamin K in the form of phylloquinone, which was injected *i*.*p*. The phylloquinone treatment during remyelination increased the levels of MK4 in the brain (**Fig 6**), as well as the total brain sulfatides content. We found a positive correlation (r = 0.702, *P-value* = 0.035) between the levels of brain MK-4 and the total brain sulfatides content. Positive correlations between the levels of MK4 and sulfatides were previously reported [12, 13, 19]. It is worth noting that the levels of MK-4 in the brain of the mice fed the 0.3% cuprizone-containing diet for 6 weeks were decreased, and this was most likely due to MK-4 being a lipid-soluble vitamin, whose volume of distribution decreases when demyelination takes place.

Interestingly, although the production of sulfatides was increased by the vitamin K treatment (**Figs 3 and 5**), when we assessed the remyelination of the corpus callosum after 1 and 3 weeks, we noticed no enhanced myelination in the vitamin K-treated mice (**Fig 4 and S1 Fig**). These results indicate that there may be other compensatory mechanisms that contribute to remyelination, when there is a vitamin K- deficiency, and consequently, there are decreased levels of brain sulfatides. However, even though myelin is still produced in the vitamin K-deficient mice, this myelin may be structurally an abnormal myelin with altered electrophysiological properties. GalCer is the exclusive precursor for the synthesis of sulfatide [11] and it has been previously shown that the mice lacking ceramide galactosyltransferase (CGT), the enzyme responsible for the production of GalCer from ceramide, do not produce GalCer or sulfatides, but they still produce myelin that contains glucocerebrosides (**Fig 8A)**, which are lipids that have not been previously identified in myelin. Interestingly, initially the myelin from those mice had a normal ultrastructural appearance, however, with age, myelin became abnormal, exhibiting splitting and vacuolization [11]. Moreover, these mice displayed conduction deficits and with age, they developed progressive hindlimb paralysis [11]. Therefore, future studies should focus on measuring the levels of glucocerebrosides and gangliosides during remyelination in mice that are either treated with vitamin K or deficient in vitamin K. Moreover, the ultrastructure of myelin that is synthetized in these mice should be analyzed. These mice could also be followed in time, to observe any functional disturbances caused by decreased levels of sulfatides since it is believed that sulfatides play important roles in myelin function and stability [11, 27, 28]. And furthermore, the localization of distinct sulfatide species in specific regions of the brain would be interesting to know and should be the focus of future studies.

To the best of our knowledge, this is the first study to show that vitamin K enhances the production of sulfatides during remyelination. Our results might provide a foundation to further study the molecular mechanism underlying remyelination in MS.

## Supporting information

S1 Fig Vitamin K treatment does not enhance the remyelination of the corpus callosum, as shown by MOG immunostaining. **A.** Representative brain coronal sections immunostained for MOG. **B.** The demyelination and remyelination of the corpus callosum was analyzed by quantifying MOG immunoreactivity. As described in *Materials and Methods* section, all images used for quantification were compared with their respective control, and the brain sections were labeled at the same time, and imaged under identical conditions. The area of the corpus the corpus callosum from the midline to below the cingulum was used to quantify the pixel intensities values using the *ImageJ* software (http://rsb.info.nih.gov/ij/). We estimated the relative intensity of MOG staining in the corpus callosum as a percentage relative to the one of the control mice, which was given the arbitrary value of 100. At least 2 sections per mouse, n = 3 or 4 mice per group have been used, and the values are expressed as mean ± SEM. **P < 0.01.
(TIF)
