# Supplementary figures and images for: Vitamin K enhances the production of brain sulfatides during remyelination
Source: PLoS One. 2018 Aug 27;13(8):e0203057. doi: 10.1371/journal.pone.0203057 (PMC6110503; doi:10.1371/journal.pone.0203057)

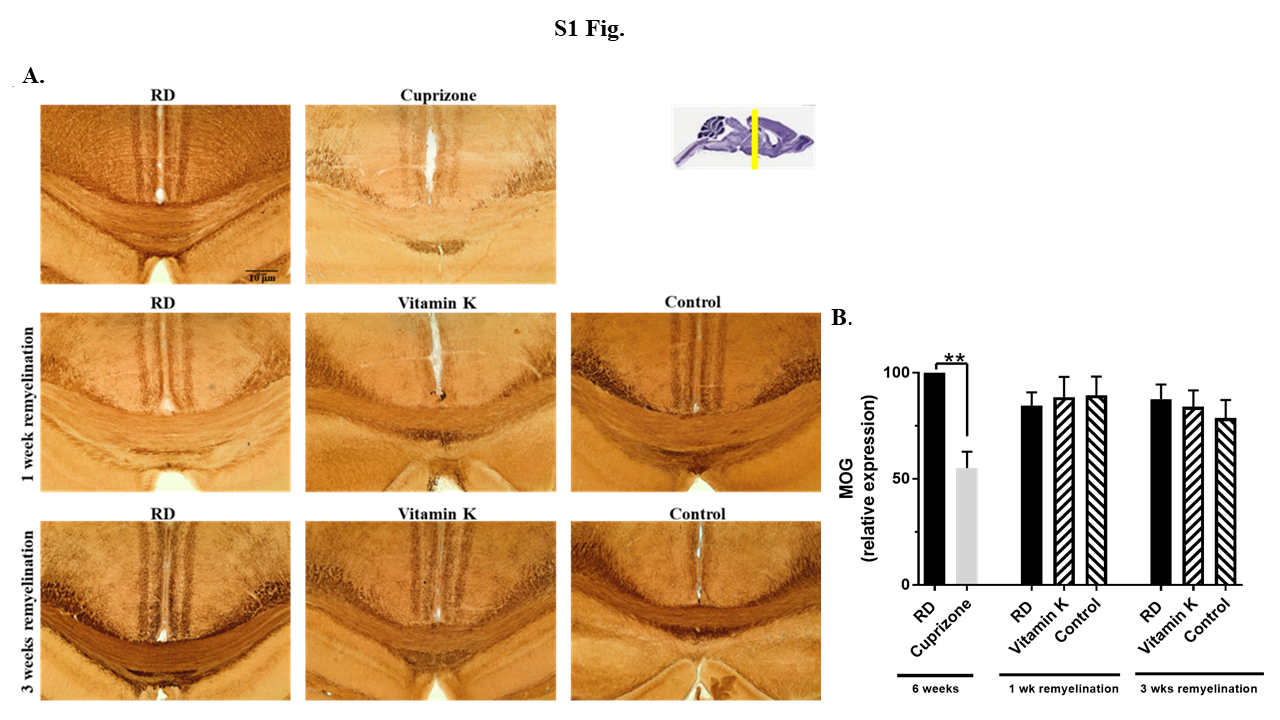

Supplement: S1 Fig — A. Representative brain coronal sections immunostained for MOG. B. The demyelination and remyelination of the corpus callosum was analyzed by quantifying MOG immunoreactivity. As described in Materials and Methods section, all images used for quantification were compared with their respective control, and the brain sections were labeled at the same time, and imaged under identical conditions. The area of the corpus the corpus callosum from the midline to below the cingulum was used to quantify the pixel intensities values using the ImageJ software (http://rsb.info.nih.gov/ij/). We estimated the relative intensity of MOG staining in the corpus callosum as a percentage relative to the one of the control mice, which was given the arbitrary value of 100. At least 2 sections per mouse, n = 3 or 4 mice per group have been used, and the values are expressed as mean ± SEM. **P < 0.01. (TIF) [file pone.0203057.s001.tif]
